# Expression of cyclooxygenase-2 and smooth muscle actin biomarkers in different subtypes of basal cell carcinoma

**DOI:** 10.3389/fmed.2025.1547778

**Published:** 2025-09-01

**Authors:** Kosar Aghajani Ilanlou, Roham Sarmadian, Sepideh Hadimaleki, Amir Vahedi, Fariba Heidari, Bahare Mehramouz

**Affiliations:** ^1^Department of Pathology, Imam Reza Hospital, Tabriz University of Medical Sciences, Tabriz, Iran; ^2^Infectious Disease Research Center, Arak University of Medical Sciences, Arak, Iran; ^3^Department of Pathology, Tabriz University of Medical Sciences, Tabriz, Iran; ^4^Department of Community and Family Medicine, Faculty of Medicine, Tabriz University of Medical Sciences, Tabriz, Iran

**Keywords:** skin cancer, basal cell carcinoma, COX-2 expression, smooth muscle actin, tumor progression

## Abstract

**Introduction:**

Basal cell carcinoma (BCC) is the most common form of skin cancer, with diverse subtypes and UV radiation as a major risk factor. Key markers like COX-2 and smooth muscle actin (SMA) are linked to tumor invasion. This study investigates their expression to improve understanding of BCC progression and treatment.

**Materials and methods:**

This *in vitro* experimental study investigated COX-2 and SMA expression in BCC subtypes from 51 paraffin-embedded tissue samples. Immunohistochemistry was performed on solid, cystic, infiltrative, adenoid, keratotic, superficial and morphea form BCCs, analyzing clinical and histological factors. Excision margin status and perineural invasion were also evaluated for their potential prognostic significance. Statistical analysis assessed marker expression and subtype relationships, considering confounding variables like the age and gender of the patients.

**Results:**

The study evaluated clinical and histological characteristics of basal cell BCC in 51 patients (mean age 64.12 ± 11.45 years, 66.7% male). Nodular BCC was the most common subtype (19.6%). SMA expression was observed in 88.2% of cases and COX-2 in 21.6%. COX-2 expression significantly correlated with invasion depth (*p* = 0.001) and recurrence (*p* = 0.007), highlighting its potential as a prognostic marker. Perineural invasion was detected in 5.9% of cases but did not show a significant correlation with invasion depth (*p* = 0.937) or tumor subtype (*p* = 0.790). No significant associations were found between protein expression and gender or lesion location. These findings support targeted diagnostic strategies. No statistically significant correlation between COX2 + staining intensity and the variables assessed, including depth of invasion, recurrence, gender, site of involvement, and tumor subtype.

**Conclusion:**

COX-2 expression significantly correlated with tumor invasion depth and recurrence in BCC, highlighting its potential as a prognostic marker and therapeutic target, while SMA showed limited relevance to tumor behavior. Although excision margin size did not show a statistically significant correlation with recurrence (*p* = 0.371), tumors with margins >0.5 cm exhibited a lower recurrence rate (11.4%), suggesting a possible impact on treatment outcomes.

## Introduction

Skin cancer is one of the most prevalent malignancies globally, encompassing types such as melanoma and non-melanoma skin cancers, including basal cell carcinoma (BCC) and squamous cell carcinoma (SCC). Among these, BCC is the most frequently diagnosed, particularly in fair skinned populations, with a lifetime risk of approximately 30% in certain demographics (1, 2). While it predominantly occurs in sun-exposed areas of the body, BCC can also develop in regions not typically exposed to sunlight. Key risk factors for BCC include fair skin, blonde hair, blue eyes, outdoor occupations, and exposure to arsenic (3, 4). According to the WHO Classification of Skin Tumors (2023), BCC subtypes are categorized into: Low-risk subtypes: superficial, nodular (including adenoid and cystic variants), infundibulocystic, and fibroepithelioma of Pinkus. High-risk subtypes: infiltrative, morpheaform (sclerosing), basosquamous, and micronodular. This classification guides clinical management due to differences in aggressiveness and recurrence potential. Although BCC is characterized by slow growth, untreated lesions can invade deeper tissues (5, 6).

Ultraviolet (UV) radiation is a critical factor in skin cancer pathogenesis, primarily due to its role in aberrant prostaglandin production, which promotes tumorigenesis. The enzyme cyclooxygenase (COX), which catalyzes the conversion of arachidonic acid to prostaglandins, is central to this process. COX exists in two isoforms: COX-1, typically expressed in normal tissues, and COX-2, which is upregulated during inflammation and cancer. COX-2 is associated with promoting cell proliferation, angiogenesis, and inhibition of apoptosis (7, 8). Evidence suggests that COX-2 inhibitors may reduce the risk of UV-induced skin cancers, with prolonged use of non-steroidal anti-inflammatory drugs (NSAIDs) showing protective effects (9–11).

Research has also identified markers such as CXCR4 and COX-2 in BCC and SCC, with correlations observed between these markers and tumor invasion depth (12). A study by El Khalawany et al. reported elevated COX-2 expression in recurrent BCC compared to primary BCC (13). Additionally, actin, a cytoskeletal protein, has emerged as a critical factor in tumor invasion and prognosis. Actin’s behavior in malignant and adjacent stromal cells suggests a pivotal role in cellular motility and invasion (14, 15). Notably, studies by Gabbiani demonstrated increased actin expression in BCC, SCC, and surrounding vasculature, linking it to tumor aggressiveness (16). Further, Sukamoto’s research revealed higher actin expression in adenoid, sclerosing, and superficial BCC subtypes, underscoring its potential in subtype differentiation and treatment planning (17). Similarly, Law’s findings on infiltrative BCC indicated an association between elevated actin levels and tumor aggressiveness (18). Pilloni et al. identified smooth muscle actin (SMA) as a marker predictive of BCC invasiveness in facial dermal regions, highlighting its prognostic value (19).

These studies collectively underline the roles of COX-2 and SMA in neoplastic processes, tumor invasion, and prognostic evaluation. Given the histological diversity of BCC and the clinical implications of these markers, a detailed investigation of their expression is crucial.

This study aims to explore the expression of COX-2 and SMA across different BCC subtypes. By analyzing these markers at the proteomic level, the study seeks to enhance understanding of their roles in invasion and disease progression. The findings may facilitate the identification of biological patterns associated with BCC progression, contributing to more targeted and effective treatment strategies for this common form of skin cancer.

## Materials and methods

### Study design and population

This research was conducted as an experimental *in vitro* laboratory study. The study population consisted of patients who visited Sina Hospital in Tabriz and were diagnosed with BCC. The patients included various subtypes of BCC, low-risk (superficial, nodular) or high-risk (infiltrative, micronodular, morpheaform) based on WHO criteria. To improve prognostic accuracy, tumors were classified into indolent (superficial, nodular) and aggressive (infiltrative, micronodular, morpheaform) subtypes. The selection of these patients aimed to investigate the relationship between the expression of COX-2 and SMA markers with clinical and histological characteristics of different BCC subtypes. Additionally, excision margins and perineural invasion were recorded to evaluate their impact on recurrence and tumor behavior. Factors such as age, gender, family history, and general health status were also considered to analyze their potential influence on the results.

### Sampling

Based on the study by Sirvikoz et al. (12) and using the ROC curve with a power analysis (*β* = 0.2, *α* = 0.05), a minimum sample size of 50 was calculated using the PASS11 software. The sampling method was purposive, based on the inclusion and exclusion criteria. Patients with a confirmed diagnosis of BCC between 2018 and 2021 at Sina Hospital were included. Exclusion criteria involved patients without sufficient tissue blocks or those whose pathology blocks were unavailable.

### Procedure

Following approval from the Medical Ethics Committee of Tabriz University of Medical Sciences, all paraffin-embedded tissue blocks from patients diagnosed with BCC in the hospital’s pathology department were included in the study. Initially, the samples were examined using hematoxylin–eosin staining to confirm the diagnosis. Subsequently, the expression of COX-2 and SMA was assessed via immunohistochemistry (IHC). The relationship between these markers and BCC subtypes was analyzed, and their expression levels were compared across the different subtypes.

### Immunohistochemistry technique

Immunohistochemistry (IHC) was employed to identify the presence and location of COX-2 and SMA proteins in tissue sections. This technique utilizes antibodies that specifically bind to target proteins, making it highly useful for cancer research. Formalin-fixed, paraffin-embedded tissues were used, and sections were prepared with a thickness of 5 μm. The slides were then deparaffinized and hydrated before undergoing antigen retrieval using heat-induced epitope retrieval (HIER) with an EDTA-Tris buffer at pH = 9. The sections were incubated with a blocking solution to prevent non-specific antibody binding, followed by overnight incubation with primary antibodies for COX-2 and SMA, diluted 1: 200. After washing, the slides were incubated with secondary antibodies and visualized using DAB chromogen. Additionally, hematoxylin and eosin (H&E) staining was performed to assess the histopathological features of each BCC subtype prior to immunohistochemical analysis. The intensity of COX2 + staining in tumor cells was evaluated based on a semi-quantitative scoring system (Table 1). Staining intensity was classified into four categories: negative (0), low (1+), moderate (2+), and high (3+). For each case, the intensity and percentage of positively stained tumor cells were assessed under high-power microscopic fields. *α*-SMA expression was assessed in both tumor cells and peritumoral stroma. Positive staining was defined as distinct cytoplasmic reactivity in myofibroblasts or tumor cells. The scoring was binary (positive/negative) due to its stromal expression pattern. This grading method is widely utilized in immunohistochemistry studies to standardize the interpretation of staining patterns and has been validated in prior research.

**Table 1:** COX2 + staining intensity scoring system.

Grid	Grade definition	Staining intensity of tumor cells	Description
0	No staining	Negative/very low	No detectable staining or in less than 30% of tumor cells.
1+	Weak staining	Low	Faint staining at least 30% of tumor cells or weak expression across all cells.
2+	Moderate staining	Moderate	Clearly visible staining at least 30% of tumor cells or moderate expression across all cells.
3+	Strong staining	High	Intense staining in more than30% of tumor cells or strong expression.

### Data analysis

Data analysis was conducted using SPSS version 27. Descriptive statistics, including mean and standard deviation for quantitative data and frequency and percentage for qualitative data, were calculated. Fisher’s exact test was used for categorical variables (e.g., COX-2/SMA expression, perineural invasion) due to small sample sizes in subgroups. Continuous variables were analyzed using Mann–Whitney. Statistical significance was set at *p* < 0.05. Age and gender were considered confounding variables, and subgroup analysis was performed accordingly.

## Results

The results showed that the age of patients was 64.12 ± 11.45 years. The gender distribution revealed a higher prevalence of BCC in men (Table 2).

**Table 2:** Distribution of gender, invasion depth, recurrence, radiotherapy history, and basal cell carcinoma subtypes in patients.

Variable			Number	Percentage (%)
Gender		Female	17	33.3
		Male	34	66.7
Invasion depth		RD	33	64.7
		Hypoderm	16	31.4
		PD	2	3.9
Recurrence		Positive	4	7.8
		Negative	47	92.2
Radiotherapy history		Positive	0	0
		Negative	51	100
Subtypes	Low risk	Nodular	10	19.6
		Superficial	1	2.0
	High risk	Infiltrative	4	7.8
		Micronodular	2	3.9
Affected area		Face	40	78.4
		Lower limb	1	2
		Scalp	7	13.8
		Ear	3	5
		Below 0.2 cm	9	17.7
		0.2–0.5	7	13.7
		Above 0.5 cm	35	68.6
Perineural invasion		Positive	3	5.9
		Negative	48	94.1

Perineural invasion was observed in 5.9% of cases (*n*: 3), but no significant correlation was found between perineural invasion and tumor subtype (*p*: 0.790). As exhibited in Table 2, 64.7% of the patients showed invasion to the reticular dermis (RD), while 31.4% had invasion into the hypodermis. Additionally, only 7.8% of patients experienced positive recurrence, indicating relatively good disease control in most cases, potentially reflecting the effectiveness of treatment protocols at the center.

**Figure 1:**
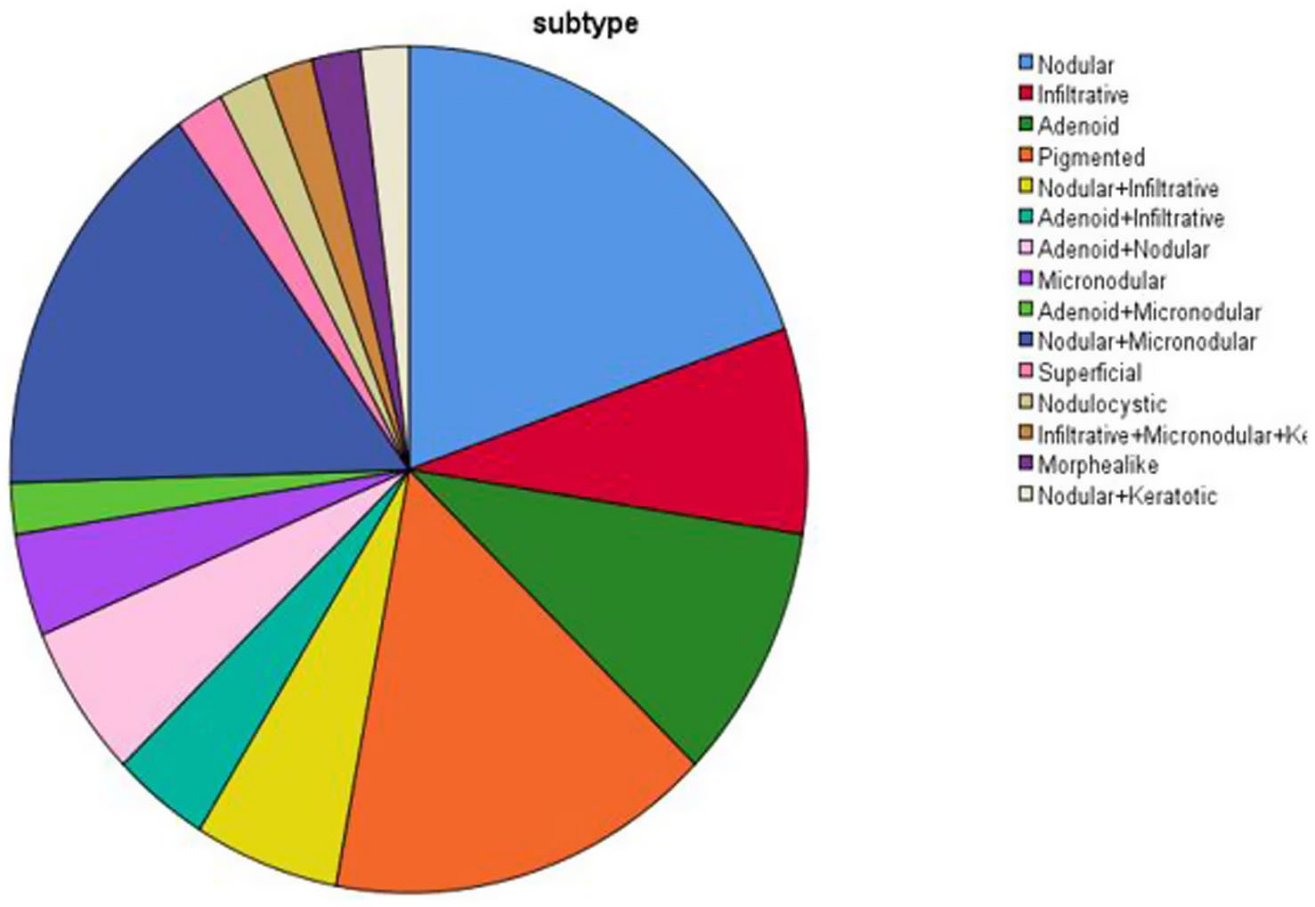
Frequency distribution of basal cell carcinoma subtypes in patients.

Regarding carcinoma subtypes, as shown in Table 2 the most frequent was the nodular type, accounting for 19.6% of cases, while the rarest subtypes (e.g., adenoid + micronodular, morphea like, infiltrative + micronodular + keratotic, nodular + keratotic) represented only 2% of cases. Figure 1 illustrates the frequency distribution of BCC subtypes among the patients.

**Figure 2:**
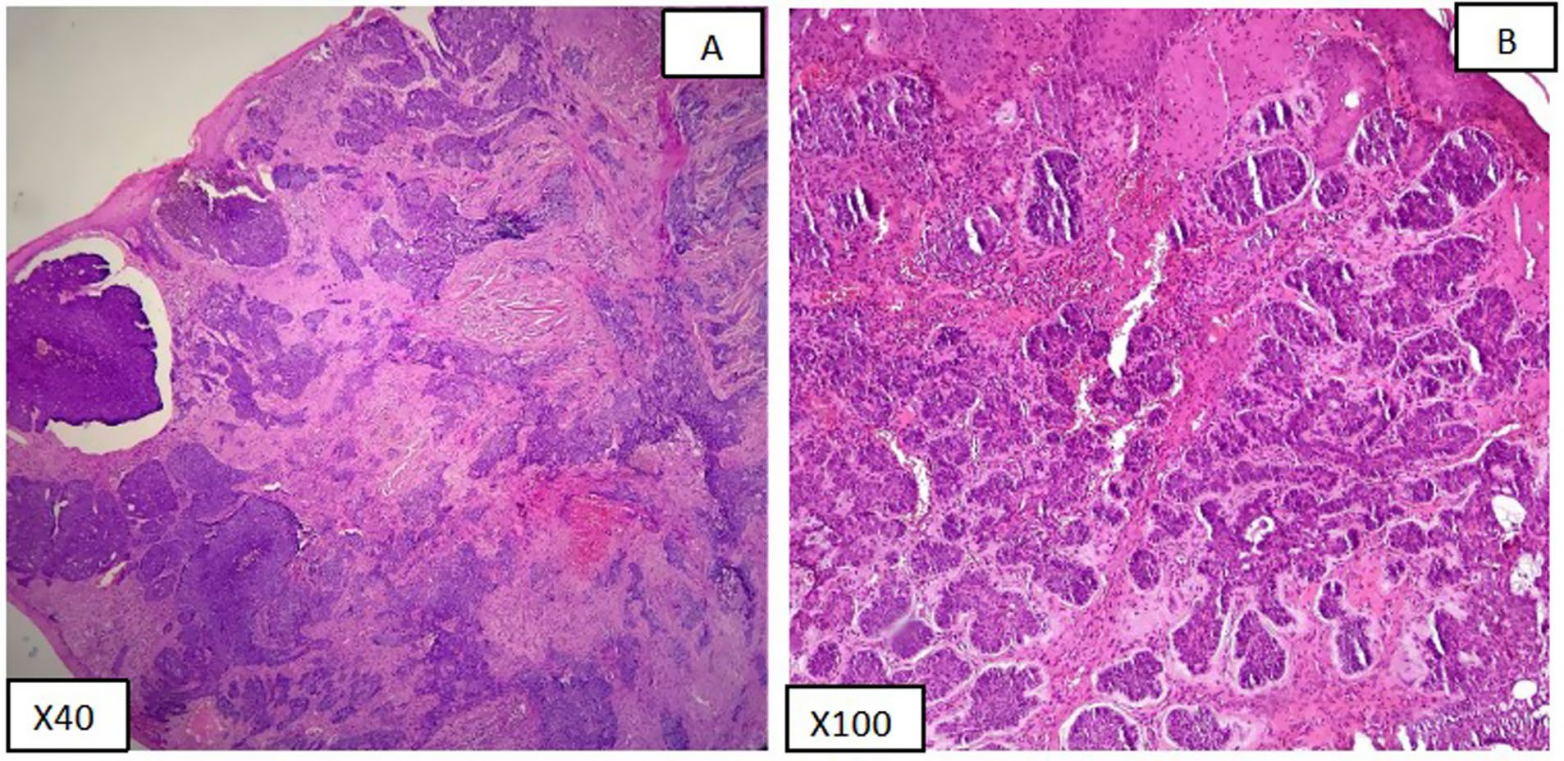
Histopathological features of BCC subtypes (H&E): **(A)** Nodular BCC (low-risk) with palisading nuclei. **(B)** Micronodular BCC (high-risk) with tumor nests <0.15 mm.

**Figure 3:**
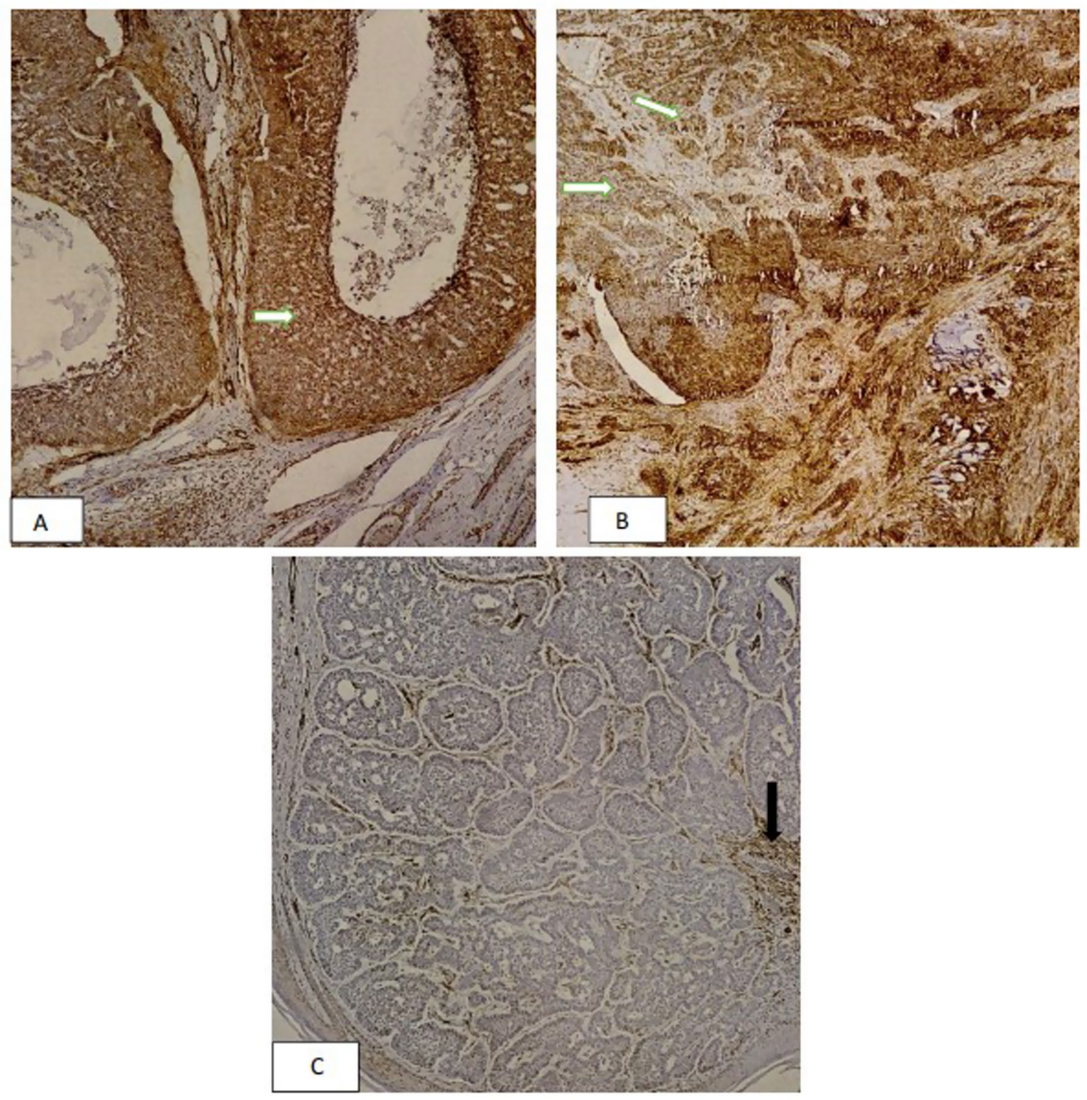
Immunohistochemical analysis of smooth muscle actin (SMA) expression in basal cell carcinoma: **(A)** Strong and diffuse SMA staining (3+) in invasive tumor nests (arrowhead) (×400) **(B)** Moderate SMA staining (2+) in tumor cells (arrowhead) (×100) **(C)** Negative SMA expression in tumor cells (×100) with preserved myofibroblasts staining (arrowheads) serving as internal positive control.

**Figure 4:**
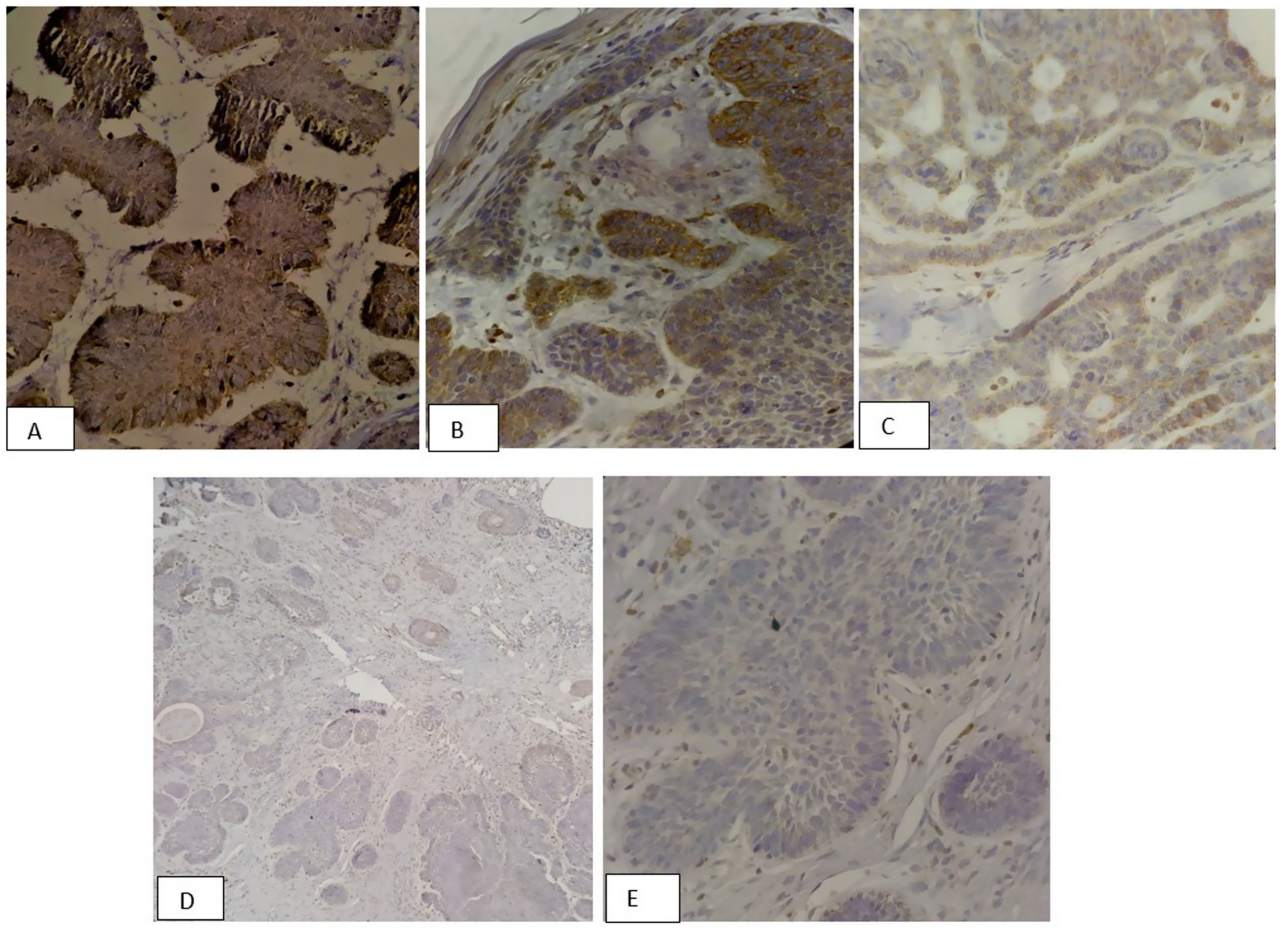
Immunohistochemical (IHC) staining patterns of COX2 in basal cell carcinoma variants: **(A)** Strong diffuse cytoplasmic COX2 expression (3+) in an aggressive micronodular BCC showing Intense staining in more than 30% of tumor cells or strong expression (×400). **(B)** Moderate COX2 expression (2+) in nodular BCC with clearly visible staining at least 30% of tumor cells or moderate expression across all cells (×100). **(C)** Focal weak COX2 positivity (1+) in adenoid BCC with faint staining in <30% of tumor cells (×100). **(D,E)** Negative COX2 expression (0) in adenoid BCC variant showing complete absence of staining (×40 and ×400).

SMA expression was positive in 88.2% of patients, underscoring the significant role of SMA as a biomarker in diagnosing and assessing BCC. On the other hand, COX2 expression was positive in 21.6% of cases. Representative H&E-stained sections highlight the histopathological characteristics of various BCC subtypes, including differences in tumor architecture and stromal response (Figure 2). These findings provide a morphological context for the subsequent immunohistochemical analysis. In Figures 3, 4, the histologic subtypes of BCC in SMA-positive and COX2-positive states can be observed.

Table 3 shows that no significant correlation was found between gender and protein expression, with *p*-value = 0.269 for COX2 and *p*-value = 0.663 for SMA, indicating that gender does not substantially influence COX2 and SMA expression levels. Similarly, statistical analysis showed no significant correlation between the carcinoma subtype and protein expression (*p* value = 0.300 for COX2 and *p*-value = 0.381 for SMA). However, a significant association was observed between invasion depth and COX2 expression (*p*-value = 0.001). This could serve as an important predictor of disease progression. SMA expression did not show a significant correlation in this regard. Additionally, a significant association was found between recurrence and COX2 expression (*p*-value = 0.007), while SMA did not show a significant association with recurrence. There was no significant correlation between lesion location and the expression of COX2 or SMA, with *p*-value = 0.386 and *p*-value = 0.488, respectively. Overall, these findings enhance the understanding of factors influencing biomarker expression in BCC and could inform more targeted diagnostic and therapeutic strategies. The results also show that tumor subtype significantly affects invasion depth. Nodular subtype showed the strongest association with invasion to RD (*p*-value = 0.01). According to Table 4, our study findings revealed no statistically significant correlation between COX2 + staining intensity and the variables assessed, including depth of invasion, recurrence, gender, site of involvement, and tumor subtype.

**Table 3:** Association between various clinical variables and COX-2 and SMA protein expression in patients with basal cell carcinoma.

Variable		SMA		*p*-value	COX2		*p*-value
		−	+		−	+*	
Gender	Female	2	15	0.663	12	5	0.269
	Male	4	30		28	6	
Subtype	Nodular	1	9	0.381	10	0	0.300
	Infiltrative	0	4		3	1	
	Adenoid	1	4		4	1	
	Pigmented	1	7		6	2	
	Nodular + Infiltrative	0	3		2	1	
	Adenoid + Infiltrative	0	2		2	0	
	Adenoid + Nodular	1	2		2	1	
	Micronodular	1	1		2	0	
	Adenoid + Micronodular	1	0		1	0	
	Nodular + Micronodular	0	8		6	2	
	Superficial	0	1		1	0	
	Nodulocystic	0	1		1	0	
	Infiltrative + Micronodular + Keratotic	0	1		0	1	
	Morphealike	0	1		0	1	
	Nodular + Keratotic	0	1		0	1	
Invasion depth	RD	6	27	0.157	31	2	0.001
	Hypoderm	0	16		8	8	
	PD	0	2		1	1	
Recurrence	positive	0	4	0.447	1	3	0.007
	Negative	6	41		39	8	
Affected area	Face	5	35	0.488	32	8	0.386
	Lower limb	0	1		1	0	
	Scalp	0	7		4	3	
	Ear	1	2		3	0	
Margin status	Below 0.2 cm	1	8	0.324	5	4	0.093
	0.2–0.5 cm	2	5		7	0	
	Above0.5 cm	3	32		28	7	
Perineural invasion		0	3	0.221	1	2	0.091

*Score +1 to +3 were grouped as positive (+) for statistical analysis.

**Table 4:** Association between COX2 + staining intensity and the variables assessed, including depth of invasion, recurrence, gender, site of involvement, and tumor subtype.

Intensity grade		+1	+2	+3	Total	*p*-value
Grade definition		Mild positive	Moderate positive	Strong positive		
COX2 intensity		8	1	2	11	0.1
Invasion depth	RD	1	0	1	2	0.724
	Hypoderm	6	1	1	8	
	PD	1	0	0	1	
Subtype	Infiltrative	1	0	0	1	0.292
	Adenoid	0	1	0	1	
	Pigmented	2	0	0	2	
	Nodular + Infiltrative	1	0	0	1	
	Adenoid + Micronodular	1	0	0	1	
	Nodular + Micronodular	1	0	1	2	
	Infiltrative + Micronodular + Keratotic	1	0	0	1	
	Morphealike	0	0	1	1	
	Nodular + Keratotic	1	0	0	1	
Recurrence	+	3	0	0	3	0.461
	−	5	1	2	8	
Gender	Male	6	0	0	6	0.084
	Female	2	1	2	5	
Affected area	Face	6	1	1	8	0.632
	Lower limb	0	0	0	0	
	Scalp	2	0	1	3	
	Ear	0	0	0	0	

In terms of tumor subtype, findings show that the nodular subtype demonstrated the greatest association with invasion to RD, but no hypodermal involvement. Other subtypes, such as infiltrative and adenoid, similarly showed cases with RD depth but had comparatively few cases extending to other layers. Overall, these results underscore a significant influence of tumor subtype on invasion depth (Table 5). Regarding gender, invasion depth was not significantly associated with gender differences (*p*-value = 0.869). Margin status was evaluated histologically, with clear margins defined as >0.5 cm and close margins as ≤0.5 cm. No recurrence was observed in tumors with margins ≤0.5 cm, compared to 11.4% recurrence in wider margins (*p* = 0.371). Additionaly our study did not find statistically significant correlation between perineural invasion and invasion depth (*p* = 0.937). However, cases with perineural invasion tended to exhibit deeper tumor infiltration, predominantly extending into the reticular dermis or hypodermis.

**Table 5:** Distribution of invasion depth of basal cell carcinoma based on gender and tumor subtype.

Variable		Invasion depth			*p*-value
		RD	Hypoderm	PD	
Gender	Female	11	5	1	0.869
	Male	22	11	1	
Type	Nodular	10	0	0	0.01
	Infiltrative	2	2	0	
	Adenoid	3	2	0	
	Pigmented	6	1	1	
	Nodular + Infiltrative	1	2	0	
	Adenoid + Infiltrative	1	1	0	
	Adenoid + Nodular	1	2	0	
	Micronodular	2	0	0	
	Adenoid + Micronodular	1	0	0	
	Nodular + Micronodular	6	2	0	
	Superficial	0	0	1	
	Nodulocystic	0	1	0	
	Infiltrative + Micronodular + Keratotic	0	1	0	
	Morphealike	0	1	0	
	Nodular + Keratotic	0	1	0	
Perineural		2	1	0	0.937

## Discussion

The results of this study offer an in-depth analysis of the expression of COX-2 and SMA biomarkers across different subtypes of BCC. The findings reveal that COX-2 expression is significantly associated with tumor invasion depth and recurrence, highlighting its potential as an essential marker for predicting disease progression and guiding therapeutic strategies. An increase in COX-2 expression correlates with greater tumor invasion depth, suggesting its active role in inflammation and tumorigenesis. This underscores COX-2’s importance as a potential biomarker for BCC diagnosis and management, enabling clinicians to make more informed treatment decisions. Notably, COX-2, as a potential therapeutic target, could contribute to the design of new drugs and the enhancement of existing treatment methods. Future research and clinical trials could further elucidate COX-2’s role in BCC development and progression and its impact on clinical outcomes, as well as assess the efficacy and safety of targeted treatments.

In contrast, the absence of a significant relationship between SMA expression and clinical features suggests that SMA may not be a reliable biomarker in this context. This indicates that SMA functions more as an indicator of muscular tissue presence within tumor samples, rather than providing insights into tumor behavior or treatment response.

The study by Pyne et al. (20) investigated the depth of BCC invasion based on tumor subtype and anatomical location, providing valuable insights into its behavior. Their findings showed that invasion depth varied significantly by subtype: nodulocystic BCCs exhibited the greatest average depth, while superficial BCCs had the shallowest. Tumors in areas with chronic sun exposure demonstrated greater invasion depth, with infiltrative BCCs in the neck region reaching an average depth of 1.8 mm. These findings align partially with our study, which also assessed invasion depth across BCC subtypes and genders. While nodular BCCs showed a significant association with invasion to the reticular dermis (RD), none extended to the hypodermis, unlike infiltrative and micronodular subtypes (Table 5). This contrasts with Pyne et al.’s report of nodulocystic BCCs exhibiting the deepest invasion. This discrepancy may reflect variations in care habits, patient age, or hormonal factors influencing tumor progression. Both studies underscore the importance of considering tumor subtype, gender, and anatomical location in the clinical management of BCC. While Pyne et al. highlighted potential gender-related differences in invasion depth, our findings emphasize subtype-specific invasion patterns.

Another study by Sivrikoz et al. (21), which investigated the expression of CXCR-4 and COX-2 in BCC and Squamous Cell Carcinoma (SCC), supports these findings. Their results demonstrated that while there were no significant differences in CXCR-4 and COX-2 expression between BCC and SCC, the expression of these markers increased with greater tumor invasion depth. Notably, COX-2 expression was significantly higher in infiltrative BCC subtypes, with all such cases exhibiting strong COX-2 expression. A significant positive correlation between COX-2 and CXCR-4 expression was also observed, suggesting these markers’ role in tumor progression. This aligns with our findings, where COX-2 was linked to deeper invasion and positive recurrence in BCC.

Ghasemi et al.’s study (22) and our research both explored the role of COX-2 in skin cancers. Ghasemi et al.’s study highlighted COX-2 as a crucial diagnostic marker distinguishing malignant melanoma from benign neoplasms, identifying a significant association between COX-2 levels and tumor stage positioning COX-2 as an effective diagnostic tool for identifying malignant melanoma. While Ghasemi’s study underscores the role of COX-2 as a key marker for melanoma diagnosis, our research suggests that examining COX-2 alongside SMA can aid in comprehending the behavior and characteristics of different BCC subtypes.

Pilloni et al. (19) demonstrated that SMA positivity is associated with aggressive features of BCC, including deeper dermal infiltration and ulceration, and can even be expressed in smaller BCCs (<3 cm). On the other hand, such an association was not observed in our study. In our study, perineural invasion was observed in a small subset of cases (5.9%) and did not show a statistically significant association with BCC subtype (*p* = 0.790). This finding aligns with previous reports suggesting that while perineural invasion is more common in aggressive tumors, its occurrence remains relatively low in BCC compared to other cutaneous malignancies. The reasons for the discrepancy between the two studies could include variations in antigen retrieval methods or sensitivity of the IHC protocol. Moreover, SMA expression may vary across BCC subtypes, and the specific subtypes included in your study may not align with those most strongly associated with SMA positivity in other studies. Furthermore, SMA expression may not be a consistent marker of aggressiveness in all populations or clinical settings, reflecting biological heterogeneity in BCC behavior.

Recognizing the role of these biomarkers could foster the development of effective therapeutic and preventive strategies. Although no significant correlation was found between excision margin size and recurrence (*p* = 0.371), we observed a trend where tumors with margins larger than 0.5 cm had a lower recurrence rate (11.4%). This observation supports previous recommendations emphasizing the importance of achieving adequate surgical margins to minimize recurrence risk (3, 4). Despite the similarities and differences between these studies, further research is needed to delve into the biological and clinical mechanisms of COX-2 and SMA and improve treatment and prevention strategies for recurrence in skin cancer patients. Our results showed no significant difference in COX-2 expression between indolent and aggressive BCC subtypes (*p* = 0.309). However, previous studies have suggested a potential role of COX-2 in promoting tumor invasion and angiogenesis (21), warranting further investigation with larger sample sizes. An enhanced understanding of factors influencing BCC progression can improve patients’ quality of life and reduce the economic burden associated with treatment. This study contributes to the existing body of knowledge on BCC and opens avenues for future research and better therapeutic approaches. While no statistically significant correlation was found between perineural invasion and invasion depth (*p* = 0.937), tumors exhibiting perineural invasion tended to infiltrate deeper layers, predominantly extending into the reticular dermis or hypodermis. This trend aligns with previous observations suggesting that perineural invasion may be a marker of more aggressive tumor behavior (18), despite its low prevalence in BCC.

This study’s primary limitations include small sample size and lack of molecular validation (e.g., qPCR) may affect generalizability of our findings, and the potential variability of IHC methods, which may influence biomarker expression due to sample quality, preparation techniques, and detection sensitivity. Additionally, while IHC results are valuable, they ideally require validation via transcriptomic approaches, such as quantitative PCR, which were not employed in this study.

## Conclusion

This study provides a comprehensive assessment of the expression of COX-2 and SMA across various subtypes of BCC, elucidating significant correlations with clinical and histological characteristics of this common skin cancer. The findings indicate a significant association between COX-2 expression and tumor invasion depth, as well as positive recurrence rates. This highlights COX-2 as a crucial marker for predicting disease progression and guiding therapeutic strategies. Specifically, the study found that COX-2 expression increased with greater tumor invasion depth, suggesting heightened enzyme activity in inflammatory and tumorigenic processes.

COX-2, a key enzyme in prostaglandin synthesis, plays a vital role in modulating inflammatory responses and promoting tumor growth. The study’s results underscore the importance of COX-2 as a potential biomarker for the diagnosis and management of BCC. While no significant correlation was found between perineural invasion and invasion depth, cases with perineural involvement exhibited a tendency toward deeper infiltration. Additionally, excision margin demonstrated a potential influence on recurrence rates, though statistical significance was not achieved. These findings suggest that integrating histopathological parameters such as perineural invasion and excision margin assessment alongside COX-2 expression could enhance prognostic accuracy in BCC. Further studies with larger cohorts are warranted to validate these observations and refine treatment guidelines. Given that COX-2 can be elevated in both early and advanced stages of the disease, assessing its levels can aid clinicians in making more informed treatment decisions. Notably, for patients exhibiting greater invasion depth, detecting elevated COX-2 levels may facilitate better risk assessment for recurrence and disease progression.

## Data Availability

The original contributions presented in the study are included in the article/supplementary material, further inquiries can be directed to the corresponding author.
